# Supplementary material for: Homeostatic responses and growth of Leymus chinensis under incrementally increasing saline-alkali stress
Source: PeerJ. 2021 Mar 1;9:e10768. doi: 10.7717/peerj.10768 (PMC7931712; doi:10.7717/peerj.10768)
Supplement: Supplemental Information 4 [file peerj-09-10768-s004.doc]

**Appendix 1**

Tests of between-subjects effects

Dependent variable: N of *L. chinensis*

| Source | Type III Sum of Squares | Df | Mean Square | F | Sig. | Partial Eta Squared |
| --- | --- | --- | --- | --- | --- | --- |
| Corrected Model | 1649.555^a^ | 24 | 68.731 | 4.879 | 0.000 | 0.745 |
| Intercept | 21797.770 | 1 | 21797.770 | 1547.333 | 0.000 | 0.975 |
| Environmental N | 925.653 | 4 | 231.413 | 16.427 | 0.000 | 0.622 |
| pH | 492.199 | 4 | 123.050 | 8.735 | 0.000 | 0.466 |
| Environmental N×pH | 147.061 | 16 | 9.191 | 0.652 | 0.821 | 0.207 |
| Error | 563.493 | 40 | 14.087 |  |  |  |
| Total | 28854.259 | 65 |  |  |  |  |
| Corrected Total | 2213.048 | 64 |  |  |  |  |

a. R Squared=0.745（Adjusted R Squared =0.593）

Dependent variable: P of *L. chinensis*

| Source | Type III Sum of Squares | Df | Mean Square | F | Sig. | Partial Eta Squared |
| --- | --- | --- | --- | --- | --- | --- |
| Corrected Model | 107.312^a^ | 24 | 4.471 | 9.514 | 0.000 | 0.835 |
| Intercept | 954.874 | 1 | 954.874 | 2031.722 | 0.000 | 0.978 |
| Environmental P | 75.434 | 4 | 18.858 | 40.126 | 0.000 | 0.781 |
| pH | 28.237 | 4 | 7.059 | 15.020 | 0.000 | 0.572 |
| Environmental P×pH | 7.496 | 16 | 0.468 | 0.997 | 0.477 | 0.262 |
| Error | 21.149 | 45 | 0.470 |  |  |  |
| Total | 1077.578 | 70 |  |  |  |  |
| Corrected Total | 128.461 | 69 |  |  |  |  |

a. R Squared=0.835（Adjusted R Squared =0.748）

Dependent variable: N:P of *L. chinensis*

| Source | Type III Sum of Squares | Df | Mean Square | F | Sig. | Partial Eta Squared |
| --- | --- | --- | --- | --- | --- | --- |
| Corrected Model | 194.391^a^ | 24 | 8.100 | 8.641 | 0.000 | 0.822 |
| Intercept | 2502.469 | 1 | 2502.469 | 2669.718 | 0.000 | 0.983 |
| Environmental N:P | 137.280 | 4 | 34.320 | 36.614 | 0.000 | 0.765 |
| pH | 42.012 | 4 | 10.503 | 11.205 | 0.000 | 0.499 |
| Environmental N:P×pH | 13.996 | 16 | 0.875 | 0.933 | 0.540 | 0.249 |
| Error | 42.181 | 45 | 0.937 |  |  |  |
| Total | 2866.727 | 70 |  |  |  |  |
| Corrected Total | 236.572 | 69 |  |  |  |  |

a. R Squared=0.822（Adjusted R Squared =0.727）

By two-way ANOVA，the results showed that N, P contents and N:P of substrate had significant effects on N, P contents and N:P of *L. chinensis*, respectively (*P*<0.01).

**Appendix 2**

In order to meet the requirement of homogeneity of variance in the two-way ANOVA, the logarithm of *H* was taken. The data after the change was homogenous (Levene's Test) and had no outliers, the residuals of which accorded with normal distribution (Shapiro-Wilk Test).

Two-way ANOVA results for the effects of elements and pH on lnH

| pH value | | Elements | |
| --- | --- | --- | --- |
| 7.5 | 1.241±0.080 b | N | 1.378±0.076 ns |
| 8.1 | 1.507±0.080 ab | P | 1.269±0.065 ns |
| 8.4 | 1.647±0.092 a | N:P | 1.486±0.068 ns |
| 8.7 | 1.384±0.080 ab |  |  |
| 9.3 | 1.109±0.113 c |  |  |
| pH | | F=4.896, df=4，*P* <0.050 | |
| Element | | F=2.678, df=2，*P* = 0.089 | |
| Element×pH | | F=0.625, df=8，*P* = 0.749 | |

Different lowercase letters represent statistically significant differences between treatments at *P*<0.05. lnH are expressed by mean ± SD. “ns” means no significant difference at *P*<0.05. The results of the two-way of ANOVA showed that there was no interaction between pH and elements on H, and pH on H had the statistically significant effect (P <0.050).
